# Prosopagnosia Due to Metastatic Brain Tumor: A Case-Based Review

**DOI:** 10.7759/cureus.55349

**Published:** 2024-03-01

**Authors:** Nora I Ivanova, Dayana M Kyuchukova, Mihael E Tsalta-Mladenov, Darina K Georgieva, Silva P Andonova

**Affiliations:** 1 Department of Neurology and Neuroscience, Medical University "Prof. Paraskev Stoyanov", Varna, BGR; 2 Second Clinic of Neurology With Intensive Care Unit and Stroke Unit, University Hospital “St. Marina”, Varna, BGR

**Keywords:** cognitive, face blindness, language disorder, cerebral tumor, tumor, brain tumor, metastatic, higher cortical functions, acquired prosopagnosia, prosopagnosia

## Abstract

Prosopagnosia, also referred to as “face blindness,” is a type of visual agnosia characterized by a decreased capacity to recognize familiar faces with a preserved ability to identify individuals based on non-facial visual traits or voice. Prosopagnosia can be categorized as developmental (DP) or acquired (AP) owing to a variety of underlying conditions, including trauma, neurodegenerative diseases, stroke, neuroinfections, and, less frequently, malignancies. Facial recognition is a complex process in which different neuronal networks are involved. The infrequent but notable higher visual-processing abnormalities can be caused by lesions of the inferior longitudinal fasciculus (ILF) in the non-dominant temporal lobe. We report a rare case of AP in a 69-year-old patient who is right-hand dominant with rectal carcinoma cerebral metastases. The patient complained of dizziness, vertigo, falls, and trouble recognizing her family members’ faces. The CT scan of the head with contrast revealed two metastatic brain lesions with vasogenic edema, as one of them was in the right cerebellar hemisphere, causing dislocation and compression of the ILF. Corticosteroids and osmotherapy were utilized as a conservative treatment approach, which resulted in the prosopagnosia being completely withdrawn. In conclusion, patients with primary brain tumors or metastatic disease rarely present with an isolated cognitive deficit such as prosopagnosia. Based on the anatomical features and the personalized approach, a conservative or surgical approach may be useful to improve higher cortical functioning.

## Introduction

Prosopagnosia is a rare disturbance of the higher cortical functions, characterized by an inability to identify faces that one is familiar with [1]. This condition can be classified as developmental (DP) or acquired (AP) due to other underlying neurological conditions. In cases where AP is the only symptom, a wide differential diagnosis must be made, excluding conditions such as stroke, neuroinfections, neurodegenerative disorders, trauma, and, less frequently, malignancies [2]. For this reason, the diagnostic workup should be based on the anatomical approach. The diagnosis could be simple in some circumstances, like vascular pathology, but it might also be more complicated in others.

Prosopagnosia is not often caused by primary or secondary brain tumors, yet in certain instances, AP may be the only clinical sign of a brain tumor. [3]. Overall, the incidence of primary brain tumors is 10.8 per 100,000 person-years [4] and more than twice as many metastatic brain tumor cases (24.2/100,000 person-years) [5]. All of the aforementioned results demonstrate the increased risk of cerebral neoplasms overall and, in turn, the higher percentage of patients who would experience either an uncommon or typical clinical presentation of a brain tumor.

We report a rare case of AP in a 69-year-old female patient with a dominant right hand due to brain metastases originating from a primary rectal malignancy. The patient also complained of dizziness, vertigo, and consecutive falls. The objective of this study is to provide a comprehensive description of this condition along with a comprehensive clinical strategy for both diagnosis and treatment.

## Case presentation

A 69-year-old female patient presented to the second clinic of neurology of the University Hospital “St. Marina,” Varna, Bulgaria, with complaints from five days ago about her inability to recognize the faces of her relatives. She was able to identify them based on their gestures, voice, and attire. A few days after the initial complaints, she complained of dizziness, vertigo, an unstable walk with side staggers, and a few falls.

The comprehensive medical history disclosed that she had undergone surgical treatment for colon cancer two years prior and that she was recently diagnosed with small-cell lung cancer, with metastases found in both adrenal glands. The patient denied having suffered a head injury, losing consciousness, having memory problems, or having any other illnesses. There were no behavioral risk factors for the patient, such as smoking or alcohol abuse.

On admission, the patient presented in fair general condition, with stable hemodynamic and vital signs, being well oriented, in an afebrile state of 36.7ºC, a regular pulse at 72 beats per minute, and a blood pressure of 120/75 mm/Hg.

A slight right-sided hemiparesis, dysarthria, and central facial palsy were observed during the neurological examination. Both the lower and upper limbs' deep tendon reflexes were normal. There were no pathological reflexes in the Babinski and Rossolimo groups. The assessment of the higher cortical functions revealed an inability to recognize familiar faces consistent with AP, with no other mental or cognitive abnormalities. The patient was not able to identify pictures of her relatives or famous people when asked to name them during her evaluation. There were no anomalies found in the initial blood samples. A detailed ophthalmological test was performed, revealing no abnormalities.

**Figure 1:**
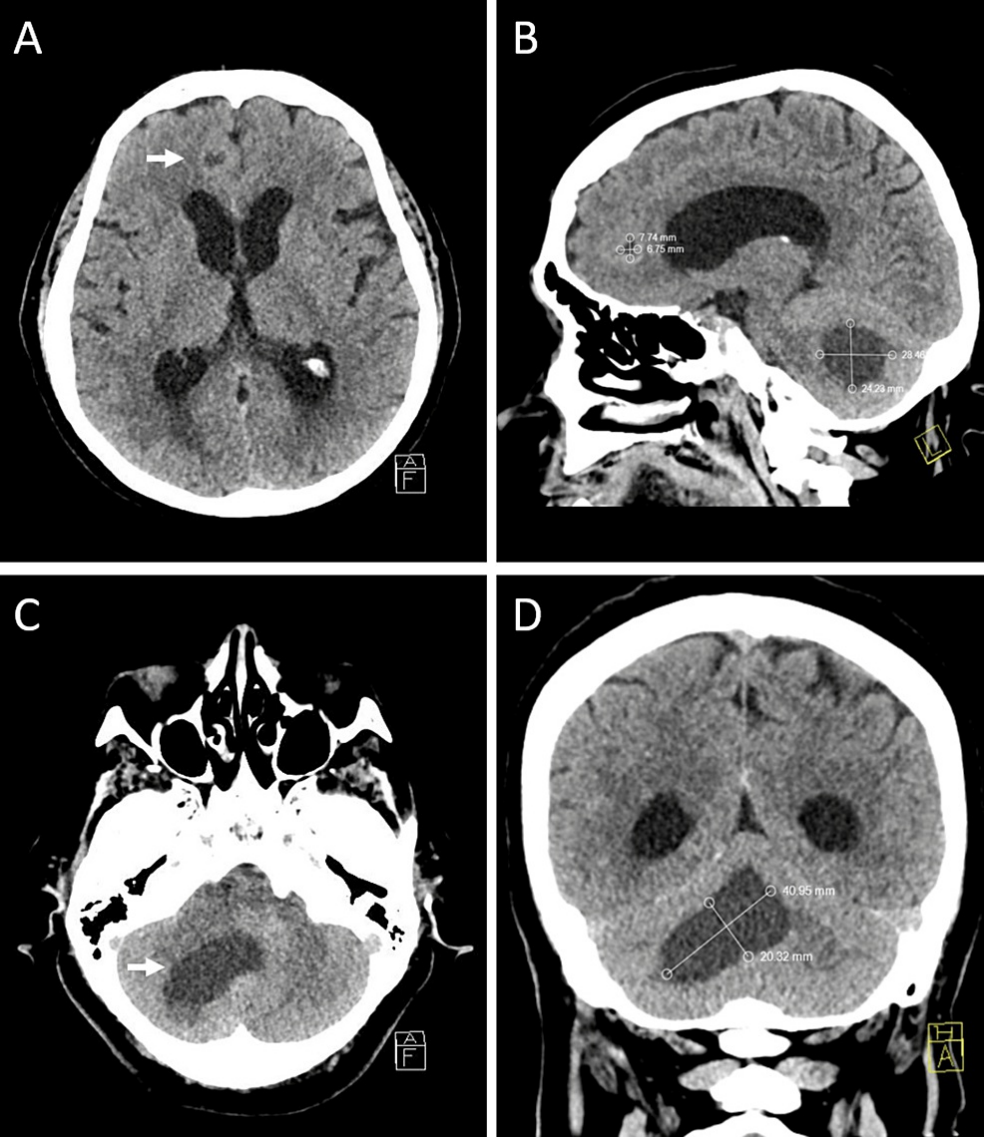
CT of metastatic brain disease (A and C) Axial views: metastatic lesion in the left frontal lobe and the right cerebellar hemisphere with perifocal cytogenic edema. (B) Sagittal view: one metastatic lesion in the frontal lobe measuring 7.74x6.75 mm and another in the right cerebellar hemisphere measuring 28.46x24.23 mm at their largest sizes. (D) Coronal view: an extensive metastatic lesion in the right cerebellar hemisphere measuring 40.95x20.32 mm at its largest, compressing the inferior longitudinal CT: computed tomography

The CT scan of the head with contrast visualized two hypodense lesions, one in the left frontal lobe and the other in the right cerebellar hemisphere. Both lesions presented with perifocal cytogenic edema and the characteristics of cerebral metastases (Figure 1). The patient was offered to have an MRI of the head, but she declined the neuroimaging procedure.

The EEG revealed that both hemispheres were irritated. The right hemisphere showed more prominent diffuse discharges of sharp and theta waves, along with brief intervals of synchronization during hyperventilation.

Hence, the patient was diagnosed with a metastatic brain disease. It was determined that the cerebellar lesion, which compressed the inferior longitudinal fasciculus (ILF) in the right non-dominant temporal lobe, was the cause of the prosopagnosia. The left frontal lobe lesion caused the later-present frontal ataxia and mild right-sided hemiparesis.

During the in-hospital stay, the patient declined surgical treatment. Despite being evaluated by a radiotherapist as a good candidate for radiotherapy, the patient declined the treatment. Therefore, a conservative treatment was initiated, including higher dosages of dexamethasone (16 mg/day), osmotherapy with intravenous mannitol along with rehydration, and antiepileptic therapy with valproic acid with titration to a target of 1000 mg/day.

The patient was released for post-hospital follow-up and care on the third day, having completely recovered from her prosopagnosia and partially from her ataxia.

## Discussion

Prosopagnosia is a rare disturbance of the higher cortical functions, characterized by the inability to recognize familiar faces and memorize new ones in the absence of intellectual, cognitive, and memory impairments [1]. This condition is often referred to as “facial blindness” or “facial agnosia,” in which individuals are able to identify a familiar only by other signs like their voice, attire, movement characteristics, and others [2].

Joachim Bodamer provided the first comprehensive description of the illness and proposed the term "prosopagnosia" in 1947, based on three personally observed cases [3]. The word comes from the Greek terms “prosopon,” which means face, and “agnosia,” which means a lack of knowledge. Due to the availability of more modern technology for measurement and imaging of the affected brain regions and neural networks during the past few decades, interest in this particular higher-cortical function disorder has grown [6].

Prosopagnosia is a clinically heterogeneous condition with two main variants. The first is the apperceptive type, in which there are difficulties in perceiving the facial structure, and the second is the associative (amnestic) type, which is characterized by difficulties remembering faces as a result of a disconnection between the perceptual and facial memory-storing areas [7].

Two types of prosopagnosia exist: DP and AP. DP is a lifelong condition that impairs a person's ability to recognize faces, while AP is due to various conditions, including stroke, neuroinfections, neurodegenerative diseases, trauma, and more rarely, tumors [2]. The prevalence in the general population of DP can reach up to 2.5%, while there is no data for the AP cases. Autosomal dominant inheritance is frequently observed in patients with DP, and it is more prevalent in children with developmental disorders such as autism and Dawn syndrome [8]. While DP cases and case series are often reported [9], descriptions of AP single cases are rare due to various underlying conditions [10].

Facial recognition is anatomically based on widely distributed neural networks encompassing several brain regions, including the hippocampus, amygdala, prefrontal cortex, and temporal and occipital cortex [2]. The superior temporal sulcus (STS), the occipital face areas (OFA), and the fusiform face areas (FFA) in the occipitotemporal gyrus comprise the majority of face-processing neuronal populations [11]. The aforementioned regions tend to be active when people observe faces, and they are associated with different modalities: OFA is associated with facial anatomy, FFA with facial identity, and STS with facial expression [12]. A white matter bundle known as the interior longitudinal fasciculus (IFL) maintains the connection between these areas [13]. IFL lesions can cause a disconnection between the frontal and posterior face neural networks, which can manifest as prosopagnosia, visual agnosia, or impaired visual memory [11].

Prosopagnosia is typically accompanied by additional neurological symptoms and is rarely caused by an isolated structural lesion [14]. A variety of medical conditions, including posterior cerebral artery strokes, hematomas, tumors, traumatic brain injuries, neurodegenerative disorders, and others, that occur primarily in the occipitotemporal cortex are known to be referred to as AP [2]. Clinical presentations of these underlying diseases can vary widely, depending on the particular cause and features of the underlying process. Additionally, they might turn in more unusual presentations, which would confound the diagnostic and treatment strategies.

In our case, prosopagnosia, ataxia, and mild right-sided hemiparesis are the combined symptoms of a metastatic brain disease that the patient is diagnosed with. The patient has two distinct lesions, one located in the left frontal lobe and one in the right cerebellar hemisphere. Unfortunately, in our case, the patient declined an MRI, which could have been essential to confirm and visualize the IFL alteration.

Utilizing functional MRI to elicit a patient's facial perception during a clinical examination can help identify whether there are damaged neural networks in the occipitotemporal region that are presenting with loss of activation [2].

Furthermore, white matter structures are typically not infiltrated by brain metastases but rather shifted [15]. In order to reduce the postoperative risk of more severe visual abnormalities and to maximize the neurological outcomes following the surgical extraction of a metastatic tumor, image-based tractography is therefore appropriate as an additional tool in the mapping of the tracts and enhancing preoperative planning [10].

Patients' prognosis and quality of life can be greatly impacted by brain metastases. When surgical intervention and radiotherapy are not an option, conservative treatment and symptom management are the only alternatives [16]. Treatment with corticosteroids (dexamethasone) and osmotherapy (intravenous mannitol), along with adequate rehydration, usually results in a decrease in intracranial pressure and peritumoral edema. In our case, due to the refusal of surgery, we conducted a conservative treatment with full recovery of the prosopagnosia. Antiepileptic therapy with valproic acid with titration to a target of 1000 mg/day. Nevertheless, in cases with asymptomatic brain metastases, corticosteroids are not recommended, and the approach to the patient should be case-based [17].

## Conclusions

Prosopagnosia is an uncommon kind of visual agnosia caused by a variety of neurological disorders, including primary and secondary brain tumors. To provide a personalized treatment plan, it is essential to identify, as quickly as possible, the precise anatomical structures affected and the underlying condition manifesting as AP. While spontaneous recovery is rare, conservative treatment with corticosteroids and osmotic medications can temporarily alleviate the neurological symptoms. Surgical treatment can be a definitive therapeutic option for well-selected patients.
